# Unlocking the bile acid universe: advanced workflows and a multidimensional library of 280 unique species

**DOI:** 10.1093/sumbio/qvag039

**Published:** 2026-09-15

**Authors:** Guozhi Zhang, Emily C Vincent, Sadie M Disselkoen, James N Dodds, Quentin DuVal-Smith, Abubaker Patan, Ipsita Mohanty, Victoria Deleray, Jian Zhang, Paul A Thiessen, Evan E Bolton, Emma L Schymanski, Pieter C Dorrestein, Casey M Theriot, Erin S Baker

**Affiliations:** Department of Chemistry, University of North Carolina at Chapel Hill, Chapel Hill, NC 27514, United States; Department of Chemistry, University of North Carolina at Chapel Hill, Chapel Hill, NC 27514, United States; Department of Chemistry, University of North Carolina at Chapel Hill, Chapel Hill, NC 27514, United States; Department of Chemistry, University of North Carolina at Chapel Hill, Chapel Hill, NC 27514, United States; Department of Chemistry, University of North Carolina at Chapel Hill, Chapel Hill, NC 27514, United States; Collaborative Mass Spectrometry Innovation Center, Skaggs School of Pharmacy and Pharmaceutical Sciences, Departments of Pharmacology and Pediatrics, University of California at San Diego, La Jolla, CA 92093, United States; Collaborative Mass Spectrometry Innovation Center, Skaggs School of Pharmacy and Pharmaceutical Sciences, Departments of Pharmacology and Pediatrics, University of California at San Diego, La Jolla, CA 92093, United States; Collaborative Mass Spectrometry Innovation Center, Skaggs School of Pharmacy and Pharmaceutical Sciences, Departments of Pharmacology and Pediatrics, University of California at San Diego, La Jolla, CA 92093, United States; National Center for Biotechnology Information, National Library of Medicine, National Institutes of Health, Bethesda, MD 20894, United States; National Center for Biotechnology Information, National Library of Medicine, National Institutes of Health, Bethesda, MD 20894, United States; National Center for Biotechnology Information, National Library of Medicine, National Institutes of Health, Bethesda, MD 20894, United States; Luxembourg Centre for Systems Biomedicine, University of Luxembourg, 6 Avenue du Swing, 4367 Belvaux, Luxembourg; Collaborative Mass Spectrometry Innovation Center, Skaggs School of Pharmacy and Pharmaceutical Sciences, Departments of Pharmacology and Pediatrics, University of California at San Diego, La Jolla, CA 92093, United States; Department of Population Health and Pathobiology, North Carolina State University, Raleigh, NC 27607, United States; Department of Chemistry, University of North Carolina at Chapel Hill, Chapel Hill, NC 27514, United States

**Keywords:** bile acids, ion mobility spectrometry, collision cross section, bile acid extraction, suspect screening

## Abstract

Microbes and bile acids are tightly intertwined, especially in the gut. While the liver produces primary bile acids from cholesterol, gut bacteria transform these into diverse secondary forms which act as powerful signaling molecules. Since bile acid changes are increasingly linked to health and disease, their accurate measurement in the gut and circulation is essential. Bile acid analytical evaluations, however, are challenging as many co-elute in liquid chromatography (LC), share identical mass spectrometry (MS) precursor masses, and produce similar tandem mass spectrometry (MS/MS) spectra. As a result, conventional LC−MS/MS workflows struggle to differentiate bile acids and have motivated the addition of orthogonal separations such as ion mobility spectrometry (IMS). Here, we assess optimal bile acid extraction parameters for stool, serum, and plasma; compare LC conditions; and assess electrospray ionization performance across polarities. Additionally, we introduce a publicly available multidimensional reference library containing LC retention times, IMS collision cross section values, and accurate precursor masses for 280 unique bile acids (264 endogenous and 16 deuterium-labeled species) including unconjugated, host-conjugated, and microbially conjugated bile acids. This multidimensional library empowers bile acid identification in complex samples and enables a more comprehensive exploration of their biological roles and disease associations.

**Sustainability Statement**

This article aligns with UN SDG3 (One Health: Good health and wellbeing) by enabling more comprehensive and accurate analytical measurements of bile acids, supporting deeper investigations into their biological roles and disease associations. These improved evaluations will also inform the development of better diagnostics and therapeutics to advance human health.

## Introduction

Bile acids are structurally diverse bioactive molecules crucial for lipid digestion, cholesterol metabolism, and host-microbiota interactions (Chiang and Ferrell 2020, Joyce and O’Malley 2022, Zhao et al. 2022, Collins et al. 2023, Fleishman and Kumar 2024). Beyond their well-established functions in dietary fat emulsification, bile acids act as potent signaling molecules that regulate metabolic pathways, immune responses, and gut microbial composition (Joyce and O’Malley 2022, Porru et al. 2022, Mancin et al. 2023, Fleishman and Kumar 2024). Disrupted bile acid metabolism is therefore implicated in metabolic syndrome, inflammatory bowel disease (IBD), and microbiota-associated dysbiosis, highlighting their potential as both diagnostic biomarkers and therapeutic targets (Heinken et al. 2019, Sultan et al. 2021, Cai et al. 2022, Yin et al. 2023, Gentry et al. 2024, Lai et al. 2024, Mohanty et al. 2024a). Recently, it was found that beyond glycine- and taurine-conjugated bile acids, gut bacteria produce microbially conjugated bile acids (MCBAs), linking molecules such as amino acids and novel neuroactive conjugates such as gamma-aminobutyric acid (GABA) to bile acids (Ridlon et al. 2014, Quinn et al. 2020, Garcia et al. 2022, Larabi et al. 2023, Mullowney et al. 2024, Van Der Kruk et al. 2025). These microbial modifications increase bile acid functional diversity and influence host neurotransmitter signaling, gut−brain axis interactions, and immune modulation (Heinken et al. 2019, Guzior and Quinn 2021, Mohanty et al. 2024b). Emerging evidence further links MCBA modifications to neurological outcomes, with altered conjugate profiles observed in extremely premature infants with brain injury (Pristner et al. 2024), reinforcing their importance. Given their biological specificity and structural diversity, accurate annotation and quantification of the many possible bile acids, particularly MCBAs, is essential for understanding their distinct biological roles in health and disease.

Despite their biological importance, identifying and evaluating bile acids remains analytically challenging due to their extensive isomeric diversity and microbial modifications (Mohanty et al. 2024b). While widely used for bile acid profiling, conventional liquid chromatography and tandem mass spectrometry (LC−MS/MS) methods often struggle to resolve isomeric species due to LC co-elution and overlapping mass-to-charge (*m/z*) ratios (Griffiths and Sjovall 2010, Humbert et al. 2012, Amplatz et al. 2017, Gu et al. 2017, Dutta et al. 2019, Zhao et al. 2022, Zhu et al. 2022, Song et al. 2023). Reliance on MS/MS fragmentation patterns alone is also typically inadequate for structural annotation since the conserved steroid core structures of bile acids often produce nearly identical fragmentation spectra, limiting differentiation of positional and stereoisomeric bile acid isomers (Mohanty et al. 2026). Furthermore, a recent study reported that 176 unique MCBAs corresponded to only 42 distinct *m/z* values, highlighting limitations of mass-based separations alone (Stewart et al. 2023). Coupling ion mobility spectrometry (IMS) with LC and MS separations (LC−IMS−MS) has emerged as a promising analytical enhancement for bile acid analyses with previous studies showing improved bile acid isomer differentiation (Zheng et al. 2017, 2018, 2019, Chouinard et al. 2018, Dodds and Baker 2019, Poland et al. 2020, Hadavi et al. 2022, Christofi and Barran 2023, Kemperman et al. 2023, Baker et al. 2024, Harris et al. 2024, Zhang et al. 2024). However, many collision cross section (CCS) values are missing for bile acids, particularly for MCBAs, limiting confident annotation in complex samples and motivating expansion of multidimensional reference measurements (Mohanty et al. 2024b, Baker et al. 2024). In many cases, as this work shows, even the bile acid structures themselves are absent from databases used for metabolite annotation.

Beyond difficulties in bile acid analytical separation and annotation, the systematic evaluation of analytical method choices remains limited including: (i) extraction methods that balance recovery and feasibility across matrices and (ii) LC conditions that separate bile acid species relevant to a given study (Humbert et al. 2012, Shafaei et al. 2021). Currently, bile acid extraction workflows differ substantially by matrix. For blood matrices, acetonitrile and methanol are widely used to extract bile acids since they efficiently precipitate proteins (Zhao et al. 2022). In contrast, fecal approaches span isopropanol-based protocols optimized for oxo-bile acids at 37°C to emerging dried fecal spot (DFS) methods that enable remote sample collection (Engevik et al. 2024). Many protocols are also labor-intensive and difficult to scale, and the limited number of head-to-head comparisons makes it unclear which parameters most strongly drive bile acid recovery (Perwaiz et al. 2002, Kakiyama et al. 2014). Furthermore, once bile acids are extracted, it is often difficult to know which LC methods will distinguish the bile acids of interest. To address these gaps, we systematically evaluated extraction solvent changes for blood and stool samples, evaluated chromatographic conditions and their trade-offs, and characterized bile acid ion formation in positive and negative MS source conditions.

Using our LC−IMS−MS workflow, we created a publicly available multidimensional bile acid reference library encompassing bile acid structures, retention times across three LC methods, experimental CCS values in positive and negative mode, and accurate masses for 280 unique bile acids. The library therefore contains 343 deprotonated features in negative ionization mode and 665 features (including sodiated, ammoniated, and protonated species) in positive mode (Tables S1 and S2). Furthermore, all bile acids were from authentic standards and mixtures as detailed in the Supplemental Information. Thus, we expect this resource to enhance bile acid identification and facilitate bile acid profiling studies across host and microbiome metabolism.

## Materials and methods

Detailed information on standard and internal standard preparation and evaluations; extraction protocols for stool, plasma, and serum; instrumental analysis; and data analysis is given in Supplemental Information and Tables S1−S8. The human samples in this study were collected in accordance with the Declaration of Helsinki, and the study was reviewed and approved by the UNC Institutional Review Board prior to recruiting any participants (approval number 16-2283 for stool samples and 15-1775 for plasma and serum). All participants provided informed consent to participate in the study, and all samples were de-identified prior to any analysis.

## Results

Evaluating analytical workflow choices is essential for confident bile acid annotation in complex matrices. Practical analytical choices that influence bile acid detectability and annotation in stool, serum, and plasma samples were investigated further in this manuscript. Each optimization step is detailed below, including the construction of a multidimensional LC−IMS−MS reference library for all bile acid ions detected in both positive and negative ionization mode.

### Bile acid extraction analyses

To support robust bile acid profiling across matrices, extraction is extremely important. Here we evaluated extraction workflows for (i) stool and (ii) serum and plasma, since matrix properties strongly influence recovery and detectability. Fig. 1 summarizes the experimental design and variables evaluated for each matrix, including extraction solvent composition and sample-to-solvent ratio. The stool, serum, and plasma extractions are detailed further below.

**Figure 1:**
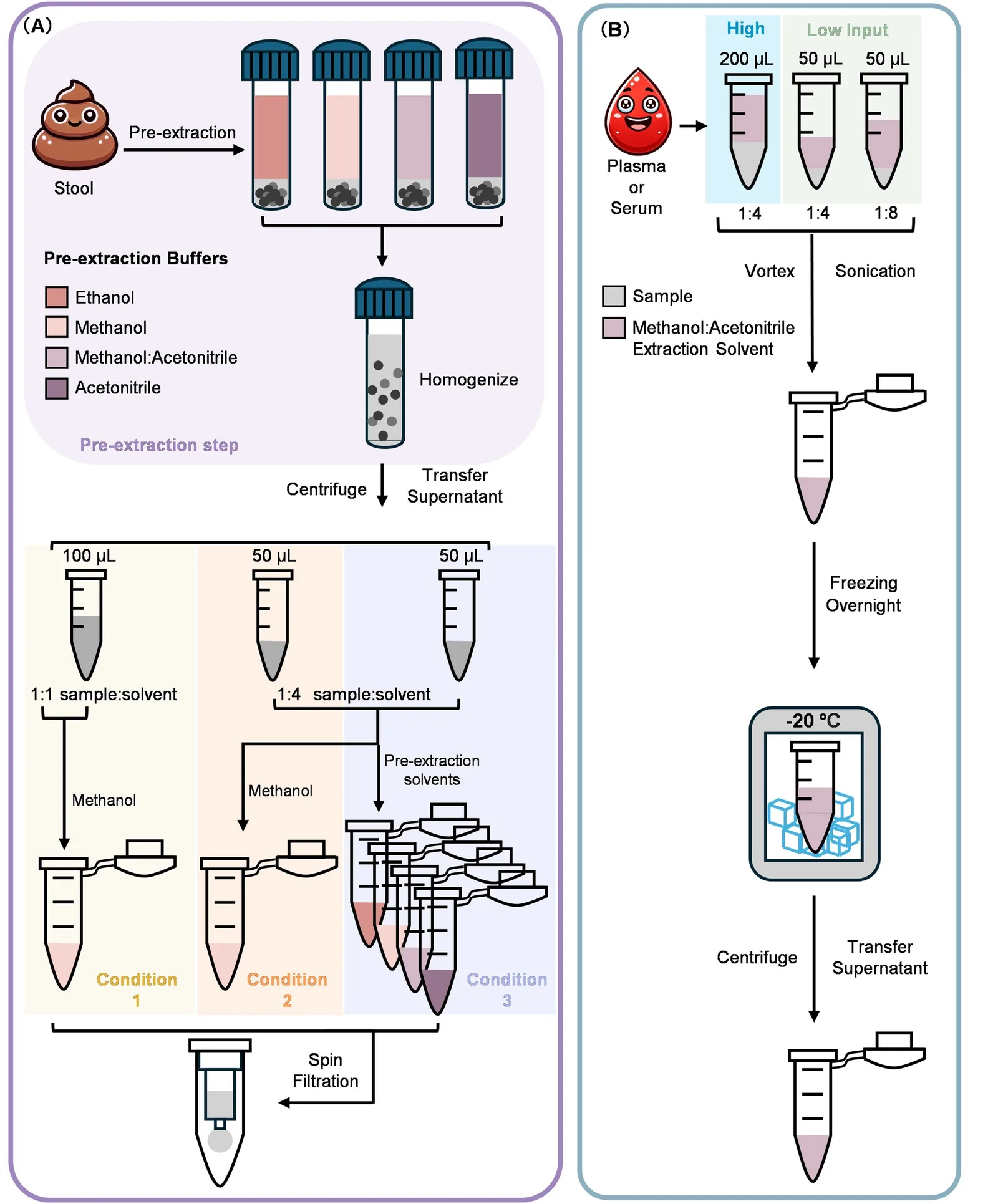
Extraction workflow and variables evaluated for stool and serum/plasma samples. (A) Stool bile acids were extracted using a two-step workflow consisting of pre-extraction step with buffers (ethanol, methanol, acetonitrile, or 1:1 acetonitrile/methanol) and then followed by a second-step dilution condition. Three dilution conditions were evaluated, including (1) 1:1 or (2) 1:4 dilution in methanol, or 1:4 dilution in the matched pre-extraction solvent. (B) Serum and plasma samples were extracted at high (200 μL) and low (50 μL) input volumes using a 1:1 acetonitrile:methanol solvent at a 1:4 sample-to-solvent ratio.

### Stool bile acid extraction

Stool bile acids are commonly extracted using solvent-based homogenization/extraction with organic solvent systems, although protocols vary widely in solvent composition and sample-to-solvent handling, systematic comparisons across these practical choices are not consistently reported. Here, we separated the effects by varying (i) pre-extraction solvent buffer composition and (ii) the second-step dilution solvent, allowing us to evaluate how these practical choices affect extraction efficiency (Fig. 1A). Briefly, ~50 mg of stool sample was extracted using a two-step workflow consisting of an initial solvent extraction (pre-extraction) followed by a second-step dilution using either methanol or the matched pre-extraction solvent. Four pre-extraction buffers (ethanol, methanol, acetonitrile, or 1:1 acetonitrile/methanol) were added directly to the raw stool in the pre-extraction step. These solvents were selected because alcohol- and acetonitrile-based extraction workflows have been previously used for bile acid extraction due to their effectiveness for metabolite solubilization, deproteinization, and compatibility with LC−IMS−MS platforms (Humbert et al. 2012, Shafaei et al. 2021, Porru et al. 2022). In the second-step, three dilution conditions were then tested: Condition 1–1:1 dilution in methanol using 100 μL supernatant and 100 μL methanol (Fig. 2A), Condition 2–1:4 dilution in methanol using 50 μL supernatant and 200 μL methanol (Fig. 2B), and Condition 3–1:4 dilution in the matched pre-extraction solvent using 50 μL supernatant and 200 μL matched solvent (Fig. 2C). The purpose of evaluating various dilution conditions was to assess optimum dilution volumes to reduce detector oversaturation without impacting detection capabilities of low-abundant bile acids. Additionally, these conditions were selected to reflect common high-throughput handling constraints and to evaluate whether transferring smaller aliquots of the initial extract (50 µL in 1:4 conditions versus 100 µL in 1:1 condition) impacted the detection or annotation of specific bile acid classes.

**Figure 2:**
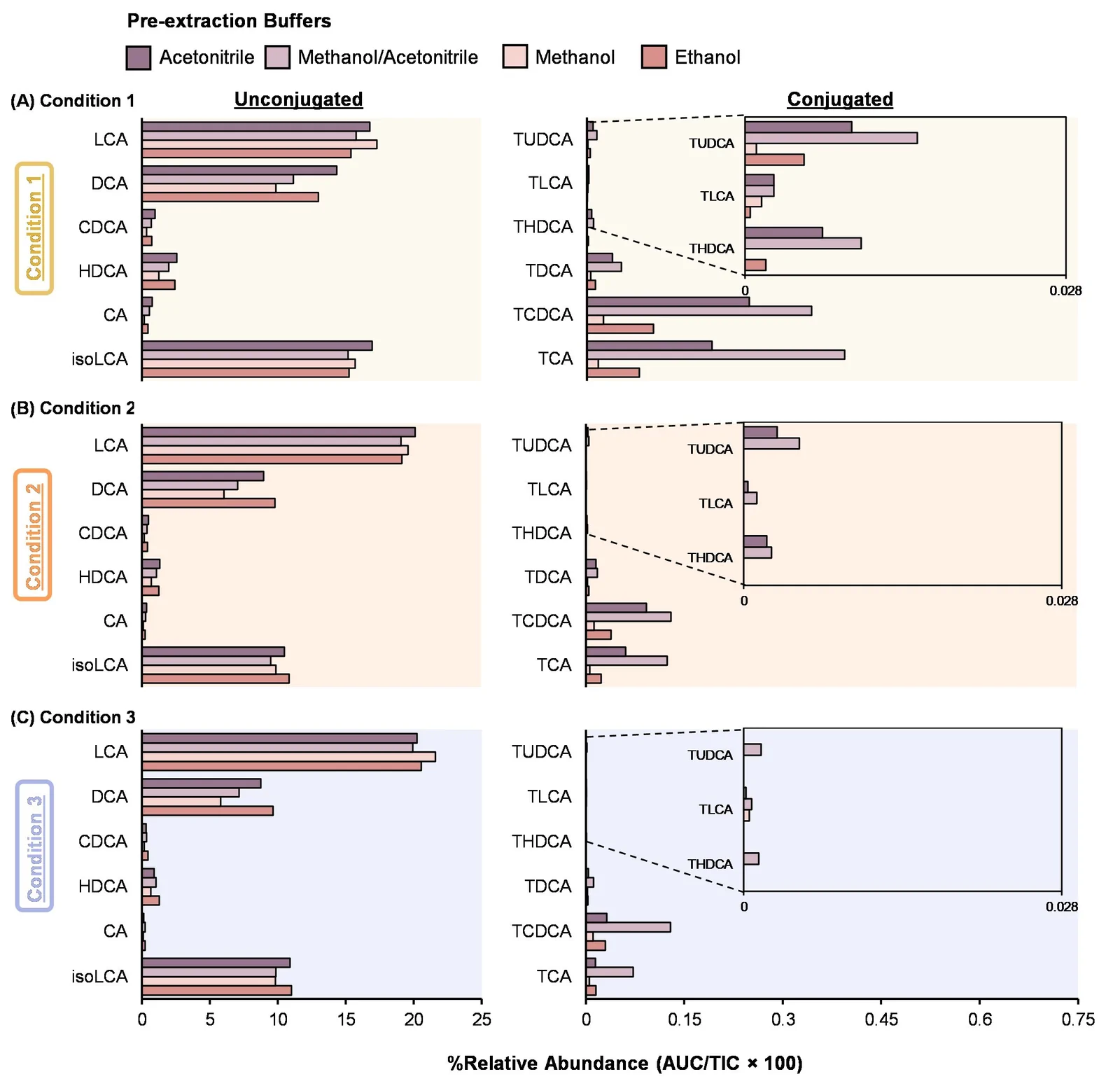
Extraction conditions on bile acid analysis from human stool samples. Bile acid extraction efficiency was evaluated using a two-step extraction workflow with different pre-extraction buffers (color-coded) and extraction ratios where: (A) Condition 1 uses a 1:1 ratio with 100 μL of pre-extraction supernatant and 100 μL of methanol, (B) Condition 2 has a 1:4 ratio with 50 μL of pre-extraction supernatant and 200 μL of methanol, and (C) Condition 3 a 1:4 ratio with 50 μL of pre-extraction supernatant and 200 μL of matching organic solvent as pre-extraction solvent. A Skyline search against the full library detected 34 bile acids, and a representative subset of 12 are shown with unconjugated (*left*) and conjugated (*right*). This subset includes lithocholic acid (LCA), deoxycholic acid (DCA), chenodeoxycholic acid (CDCA), hyodeoxycholic acid (HDCA), cholic acid (CA), isolithocholic acid (isoLCA), as well as their taurine conjugates. Percent relative abundance was calculated based on average area under the peak/total ion current × 100. This analysis was only tested in duplicate due to sample limitations.

For unconjugated bile acids, no major differences were observed between the four solvent types in the pre-extraction buffers (Fig. 2A−C, left). Across the second-step dilution conditions, most unconjugated bile acids showed modest changes in signal intensity, with up to a 53% decrease in relative abundance in Conditions 2 and 3 compared to Condition 1. This was also true when using the larger volume (100 μL vs. 50 μL) of pre-extraction supernatant. One exception was lithocholic acid (LCA), which exhibited up to a 25% increase in relative abundance under Conditions 2 and 3. Under the LC conditions used in this study, LCA elutes approximately one minute later than the nearest bile acid, resulting in chromatographic isolation relative to other bile acids in the library. Consequently, the increased signal intensity observed under Conditions 2 and 3 may reflect reduced matrix interference associated with the lower transferred extract volume, consistent with dilution-based approaches for reducing matrix effects in LC−MS (Ferrer et al. 2011). In contrast, the conjugated bile acids showed greater changes with extraction conditions (Fig. 2A−C, right). For taurine-conjugated bile acids, choice of pre-extraction buffer led to measurable differences in extraction efficiency. Specifically, using a 1:1 methanol: acetonitrile buffer in the pre-extraction step resulted in up to a 23-fold increase in relative abundance across the three dilution conditions. Furthermore, conjugated bile acid losses were observed in both Conditions 2 and 3, particularly when methanol or ethanol were used as pre-extraction buffers. Using the matching organic solvent in Condition 3 also caused more signal reduction than Condition 2 (Fig. 2B and C). For the glycine-conjugated bile acids, the extraction efficiency followed a similar trend as observed for taurine-conjugated bile acids (Fig. S1). The improved performance of the combined acetonitrile: methanol extraction solvent may reflect broader polarity coverage and improved solubilization of conjugated bile acids relative to single-solvent systems (Lepoittevin et al. 2023). These effects may be particularly relevant for microbially conjugated bile acids, which are structurally diverse based on their amino acid conjugates and may exhibit distinct extraction behavior relative to canonical species. Thus, among the stool extraction conditions tested, 1:1 acetonitrile:methanol pre-extraction followed by the 1:1 methanol dilution in Condition 1 provided the most consistent overall profile, balancing recovery with detection coverage (Fig. 2A).

### Serum and plasma bile acid extraction

Since the stool matrix is particulate-rich and highly variable, we performed a broad comparison of solvent compositions and second-step dilution schemes to identify conditions that preserve both unconjugated and conjugated bile acids. In contrast, serum and plasma are protein-rich, and bile acids are typically quantified from the post-precipitation supernatant following organic-solvent protein precipitation. Thus, rather than optimize the 1:1 acetonitrile:methanol solvent, which is widely used for serum/plasma protein precipitation, we focused on sample input volume as a practical constraint that often limits study designs. We also wanted to include both human serum and plasma because they can differ in matrix composition (e.g. clotting factors and anticoagulants), which may impact recovery.

To extract the bile acids from the human control serum and plasma, the 1:1 acetonitrile:methanol extraction solvent was fixed at 1:4 sample:solvent ratio (Fig. 1). For example, both 50 μL and 200 μL starting volumes of serum and plasma were utilized to understand recovery differences. This resulted in either 200 μL of solvent added to the 50 μL samples or 800 μL of solvent added to the 200 μL samples. Across both serum and plasma, the higher input volume (200 μL) improved detectability of low-abundance conjugated bile acids (Fig. 3A and B, right). However, Welch’s t-tests with false discovery rate adjustment showed no statistically significant differences in relative abundance between the 200 µL and 50 µL extraction conditions across the evaluated subset (*p*_adj_ > 0.05). One notable exception was tauroursodeoxycholic acid (TUDCA), which became undetectable in serum at 50 μL under the same 1:4 ratio (Fig. 3B). This observation is not unexpected, as humans exhibit a strong preference for glycine conjugation relative to taurine conjugation, with taurine-conjugated bile acids representing as little as 10% of the conjugated bile acid pool of some individuals (Falany et al. 1994, Solaas et al. 2000). Consequently, TUDCA concentrations may fall below analytical limits of detection in low-volume extractions (Sathe et al. 2020, Duszka et al. 2022). These findings suggest that extraction solvent ratios and input volumes should be selected based on primary analytical objectives, particularly when targeting low-abundance, clinically relevant bile acids. Overall, these results suggest that smaller input volumes can still support broad bile acid profiling but may reduce detection and annotation confidence for low-abundance conjugated bile acids. For glycine-conjugated bile acids, the extraction efficiency followed a similar trend to the taurine-conjugated bile acids, reflecting their comparable extraction behavior (Fig. S2).

**Figure 3:**
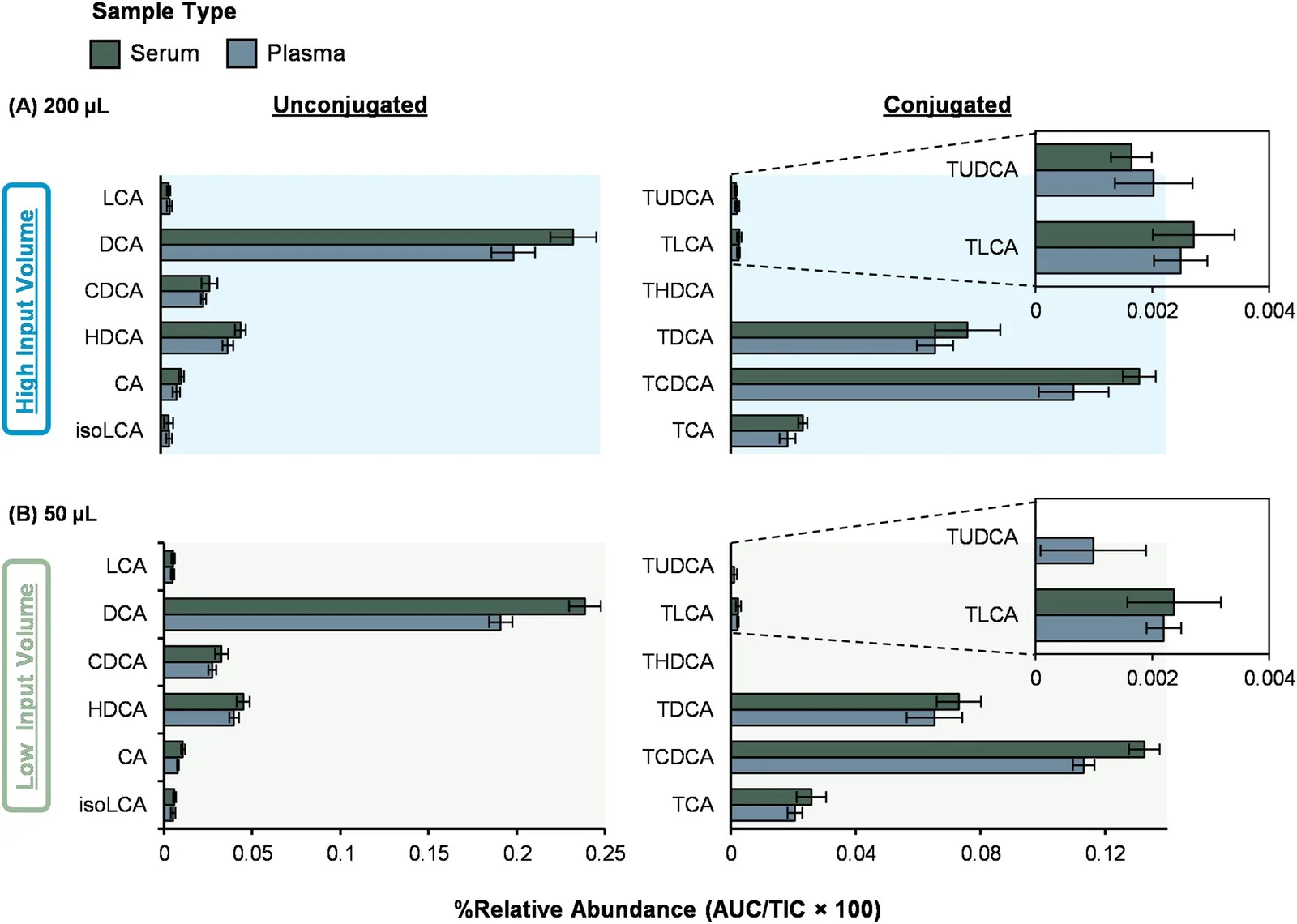
Extraction conditions for human serum and plasma samples. Bile acid extraction performance was evaluated at a fixed 1:4 sample-to-solvent ratio for both (A) 200 μL and (B) 50 μL serum and plasma volumes using 1:1 acetonitrile/methanol. A Skyline search against the full library detected 34 bile acids, and a representative subset of 12 are shown with unconjugated (*left*) and conjugated (*right*). This subset includes lithocholic acid (LCA), deoxycholic acid (DCA), chenodeoxycholic acid (CDCA), hyodeoxycholic acid (HDCA), cholic acid (CA), isolithocholic acid (isoLCA), as well as their taurine conjugates. Percent relative abundance was calculated based on average area under the peak/total ion current × 100 (n = 3 biological replicates).

NIST reference serum and plasma were also processed alongside the study samples for all extraction conditions. In all cases, they showed similar results to the control human samples (Fig. S3). We also tested an exploratory higher-dilution condition (1:8 with 50 μL) to probe the effect of increased dilution at low sample input (Fig. S4). The 1:8 condition suggested further loss of taurine-conjugated bile acids and is not recommended. Thus, we recommend the 1:4 sample-to-solvent extraction using 1:1 acetonitrile:methanol and 200 μL input when available. When sample is limited, 50 μL at the same 1:4 ratio is a practical alternative but may reduce detection and annotation confidence for lower abundance conjugated bile acids. Prior bile acid extraction workflows for plasma and serum employ acetonitrile-based extraction solvent paired with solid phase extraction cleanup (Humbert et al. 2012, Shafaei et al. 2021, Zhang et al. 2024). In contrast, the recommended workflow uses a simplified 1:1 acetonitrile:methanol extraction without SPE, enabling consistent processing across stool, serum, and plasma while maintaining broad signal coverage across diverse bile acid classes, including lower-abundance conjugated species. Although direct quantitative comparison to prior studies is limited by differences in performance metrics, these results collectively support the utility of solvent-based extraction strategies for bile acid profiling.

### LC separations

Chromatographic separation is critical for confident bile acid annotation as mass spectrometry analysis alone often cannot distinguish bile acid isomers with identical precursor *m/z* values and similar fragmentation. Here, we evaluated the effects of mobile phase pH and stationary phase chemistry using two reverse-phase columns and two bile acid mixtures: (i) a Cayman 18-bile acid mixture containing unconjugated bile acids and glycine- and taurine-conjugated species (catalog no. 33505) and (ii) 22 synthetic amino acid-conjugated cholic acids (AA-CA) as representative MCBAs (Figs 4 and 5). The three sets of mobile phase compositions and their gradient programs are provided in the Supplemental Information and Table S4. Retention times for bile acids detected under the three LC conditions are also provided in Tables S1 and S2. Because experiments were acquired across different days, absolute signal intensities are run-specific and intended for comparison within figures rather than across figures (Figs 4−6).

**Figure 4:**
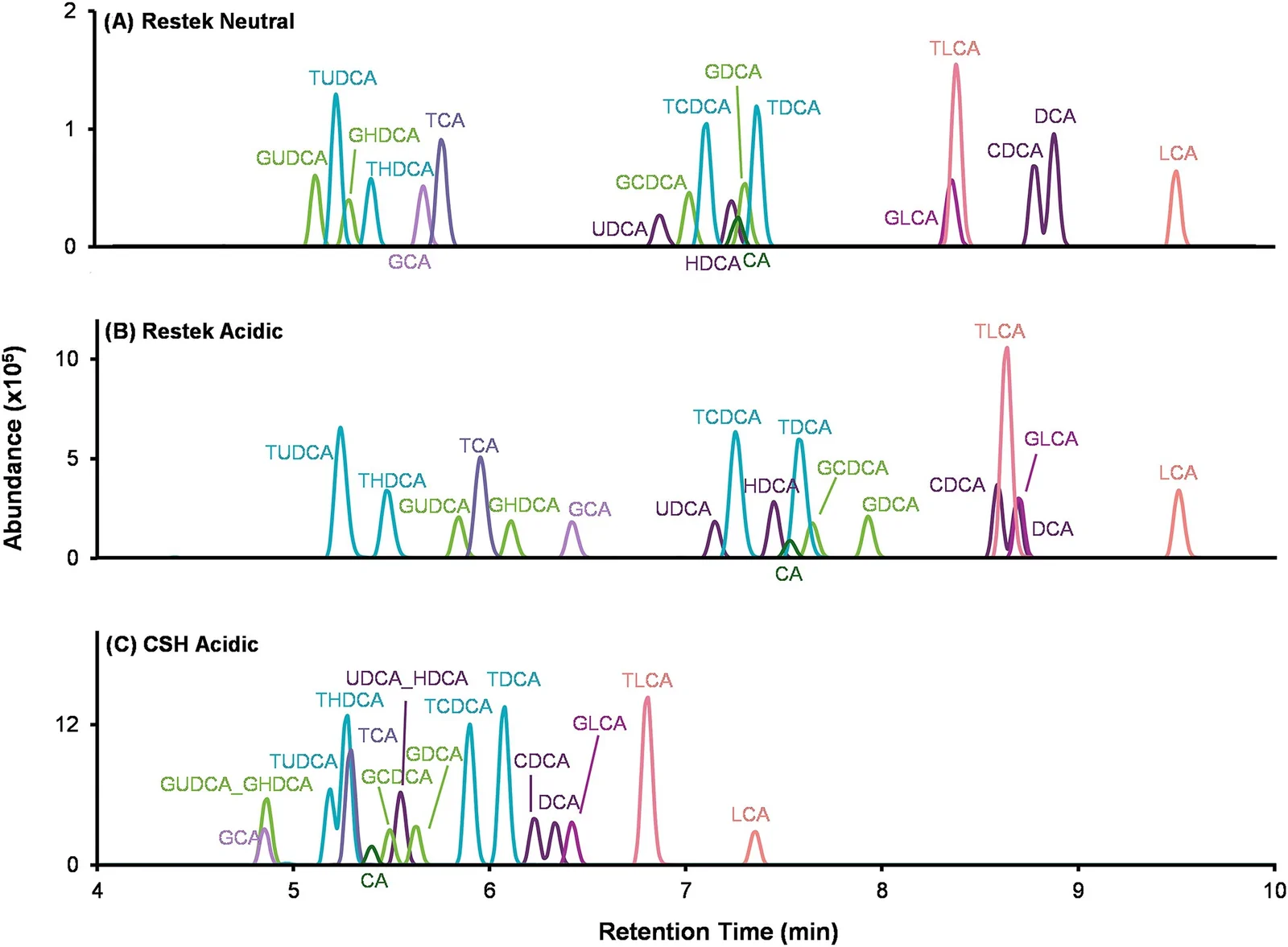
LC effects on bile acid separation. Three LC methods were evaluated using an 18 bile acid standard mixture to assess the effects of mobile-phase pH and stationary phase chemistry. (A) Restek Raptor C18 column with a neutral mobile phase. (B) Restek Raptor C18 column with an acidic mobile phase containing 0.1% formic acid. (C) CSH C18 column with the acidic mobile phase.

**Figure 5:**
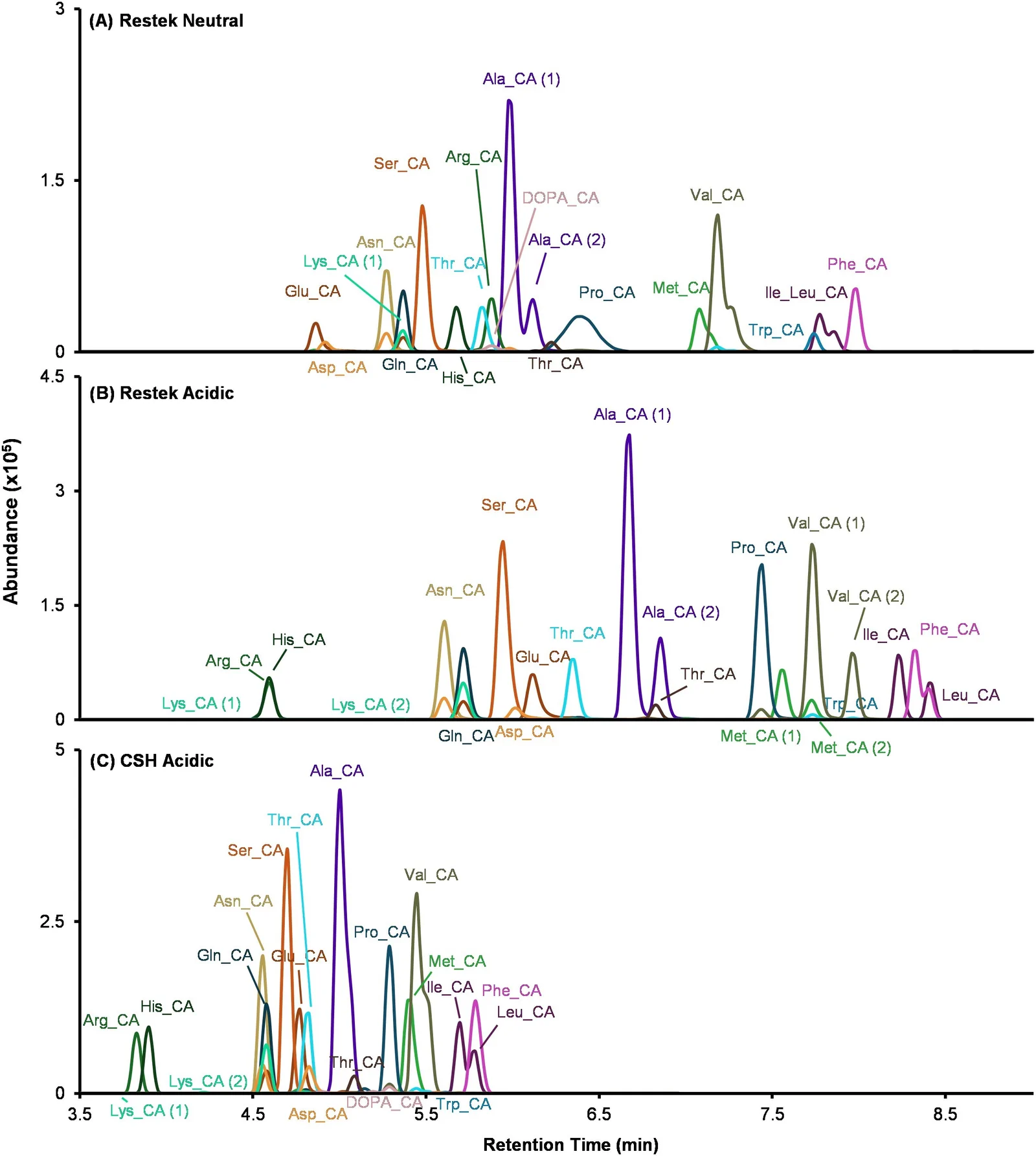
LC effects on separation of amino acid-conjugated cholic acids. Three LC methods were evaluated using 22 amino acid-conjugated cholic acids to compare separation under different mobile-phase pH and stationary phase chemistries. (A) Restek Raptor C18, neutral mobile phase. (B) Restek Raptor C18, acidic mobile phase containing 0.1% formic acid. (C) CSH C18, acidic mobile phase.

**Figure 6:**
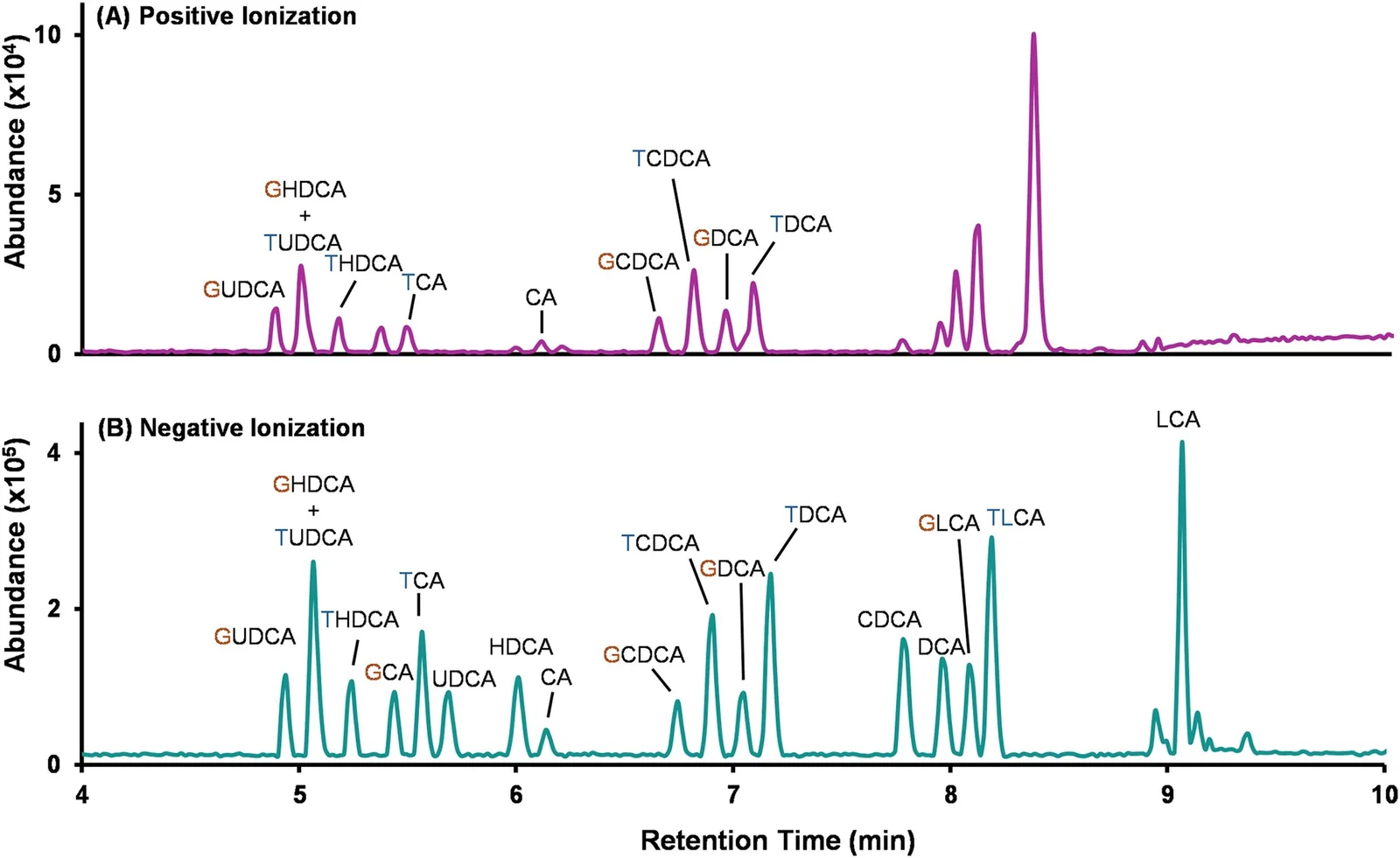
Effect of ionization mode on bile acid detection. Base peak chromatograms acquired in positive (top) and negative (bottom) ionization for the 18 bile acid standard mix. Higher signal intensity and a more uniform response were observed in negative ionization mode. Peaks are labeled by bile acid identity. In positive mode, several bile acids in the 18-component mixture did not produce a detectable signal under the acquisition conditions used. Unlabeled peaks reflect additional signals that do not correspond to the 18 standards, including background ions and other mixture-related features.

We first tested three LC methods using the Cayman standard mix to isolate the effects of mobile phase pH and column chemistry (Fig. 4). Briefly, as noted in Supplemental Information, Method 1 used a Restek Raptor C18 column (1.8 µm particle size, 50 × 2.1 mm) with a neutral mobile phase (Fig. 4A); Method 2 used the same Restek column with an acidic mobile phase, adding 0.1% formic acid to assess pH effects on retention and selectivity (Fig. 4B); and Method 3 used the same acidic mobile phase on a Waters ACQUITY Premier charged surface hybrid (CSH) C18 column (1.7 µm particle size, 2.1 × 100 mm) to evaluate whether additional charge interactions on the stationary phase under acidic conditions improve separation of bile acids (Fig. 4C). This design isolates the effects of mobile phase pH (Fig. 4A vs. Fig. 4B) and stationary phase chemistry under acidic conditions (Fig. 4B vs. Fig. 4C). On the Restek column, the acidic mobile phase (referred to as “Restek acidic” method) reduced peak overlap compared to the neutral mobile phase (“Restek neutral” method), consistent with pH-dependent changes in bile acid ionization, retention, and selectivity on the same stationary phase. Under acidic conditions, suppression of bile acid deprotonation likely altered hydrophobic interactions with the stationary phase, improving separation of several isomeric bile acid species (Zhang et al. 2022). By comparison, the CSH column with an acidic mobile phase reduced co-elution for some bile acids that were observed with the Restek methods (e.g. GLCA with TLCA (*Restek neutral*), and GLCA with DCA (*Restek acidic*)), but reduced separation for the isomer pair UDCA and HDCA and their glycine and taurine conjugated forms. Thus, each column has benefits and limitations.

Since MCBAs include diverse conjugation chemistries, we expected their chromatographic behavior to be sensitive to mobile phase pH and column chemistry. We therefore analyzed a set of 22 AA-CA species across the same three LC methods (Fig. 5), comparing peak overlap and qualitative separation patterns across methods and monitoring column performance during repeated injections. Across methods, mobile phase pH altered AA-CA elution order and selectivity. For workflows that require neutral mobile phase conditions for method compatibility or to reduce column stress under extended use, the Restek C18 column with a neutral mobile phase (Fig. 5A) separated many bile acids. The Restek C18 column with an acidic mobile phase (Fig. 5B), however, improved separation (e.g. isoleucine- and leucine-conjugated cholic acid (Ile/Leu-CA)), supporting more detailed structural analysis of the bile acids. The CSH C18 column with an acidic mobile phase (Fig. 5C) produced shorter elution times for the amino acid-conjugated cholic acids, with AA-CA eluting before 6 min, thereby reducing overlap with unconjugated bile acids (Fig. 5C vs Fig. 4C). Additionally, the shorter LC gradient time supports this method as an efficient option for higher-throughput analyses. During extended use, gradual increases in backpressure and minor retention time shifts were observed, suggesting that incorporation of a guard column may improve long-term method stability.

Across the 18 bile acid standard mixture, acidic conditions generally increased detection signal under our tested methods, whereas AA-CA signals were broadly similar across neutral and acidic conditions. Because chromatographic selectivity and elution order also changed between conditions, we describe these differences as method-dependent rather than attributing them solely to intrinsic ionization efficiency. Therefore, selection of mobile phase and column should balance isomer separation and long-term robustness and be tailored to specific study objectives and throughput requirements. Thus, our observations are that the Restek C18 column with acidic mobile phase conditions provided improved separation for several targets but exhibited gradual increased in backpressure and minor retention time drift under the operating conditions used in this study. As these effects may reflect several contributing factors, including matrix buildup or solvent-related effects, they may be interpreted as practical considerations associated with extended analysis of complex biological samples. For studies requiring neutral pH conditions or operational stability over extended injections, the Restek C18 column with a neutral mobile phase may be the preferred choice, despite its lower separation performance. For large-scale studies where maximizing chromatographic separation and reducing peak co-elution is not the primary objective, the CSH C18 column under acidic mobile phase offers a higher throughput option and maintained more stable backpressure, indicating better tolerance to acidic operation.

### Ionization mode selection

Ionization polarity strongly influences bile acid detectability and annotation (Fig. 6). We compared positive and negative polarities with ESI using the Cayman Standard Mixture containing 18 bile acids which include unconjugated and taurine- and glycine-conjugated species (catalog no. 33 505). These comparisons were performed using Method 1 on the Restek C18 column with a neutral mobile phase to provide a consistent baseline condition for evaluating polarity-dependent ion formation. Under the tested conditions, negative ion mode exhibited higher signal intensities (approximately eight-fold greater) across most tested bile acids, consistent with improved detectability in negative mode. Moreover, negative mode provided a more uniform ionization response and cleaner background in the base peak chromatogram across bile acid species, which is advantageous for profiling workflows that aim to capture both high- and low-abundance species.

In positive mode, bile acids formed protonated, sodiated, and ammoniated ions ([M + H]⁺, [M + Na]⁺, and [M + NH₄]⁺), which can provide complementary evidence for annotation when matched to the reference library. However, because adduct distributions vary by structure and acquisition conditions, signal can be distributed across multiple ion forms, which complicates consistent quantification when different adducts dominate for different bile acids. This resulted in not all bile acids in the standard mixture producing a detectable signal and additional non-target features being observed in the base peak chromatogram (Fig. 6A). Therefore, we prioritized negative mode for primary profiling and quantification, and we treat positive mode as a complementary polarity for additional confirmation when needed. Tables S1 and S2 list features detected in each polarity and their observed ion forms, enabling users to determine whether a target bile acid is detected for a given mode and whether multiple adducts can be used to support annotation.

### Multidimensional bile acid library

To support bile acid annotation and structural characterization, we created a multidimensional LC−IMS−MS reference library containing LC retention times, experimental drift time IMS CCS values in nitrogen (^DT^CCS_N2_), and accurate masses for 280 unique bile acids with each ion form and adduct annotation detected (Tables S1 and S2). Chemical structure information and identifiers for each bile acid are also included in Table S3. While other CCS repositories have focused on limited subsets of bile acids, individually focusing on canonical unconjugated bile acids, oxidized bile acids, glycine/taurine conjugates, and MCBAs (Poland et al. 2020, Stewart et al. 2023, Baker et al. 2024), we provide a comprehensive resource that provides broad coverage across endogenous, host-conjugated, and microbially conjugated classes. In total, 18.9% (53 of 280 unique bile acids) were structures that were not yet present in PubChem (new CIDs 177 860 373–177 860 425 were created upon deposition of the structures) and are unique to this multidimensional library. The library comprises 343 deprotonated features in negative ionization mode and 665 features (including sodiated, ammoniated, and protonated species) in positive mode. When multiple features corresponded to the same bile acid identity, we assigned suffixes based on separation dimension: where (1) or (2) indicate LC-resolved features separated by retention time, whereas (a) or (b) indicates features separated primarily by IMS. This convention is used consistently throughout Tables S1 and S2. To illustrate trends observed in the bile acids, CCS versus *m/z* relationships for the library are shown for negative mode (Fig. 7A) and positive mode (Figs. 7B and S5 for different ion types). Across ionization modes and adduct types, bile acids show distinct groupings by conjugate class. For example, glycine- and taurine-conjugated bile acids form separable clusters in CCS versus *m/z* space. Additionally, incorporation of CCS as a molecular descriptor improves discrimination of biologically relevant canonical and microbially conjugated bile acids. For example, while lithocholic acid and isolithocholic acid are only partially resolved by LC (0.5 min retention time difference), they remain distinguishable in the IMS dimension through a 5.4 Å² CCS difference. Thus, this multidimensional library should improve discrimination of structurally similar bile acids, including isomers and amino acid-conjugated species, in complex biological samples.

**Figure 7:**
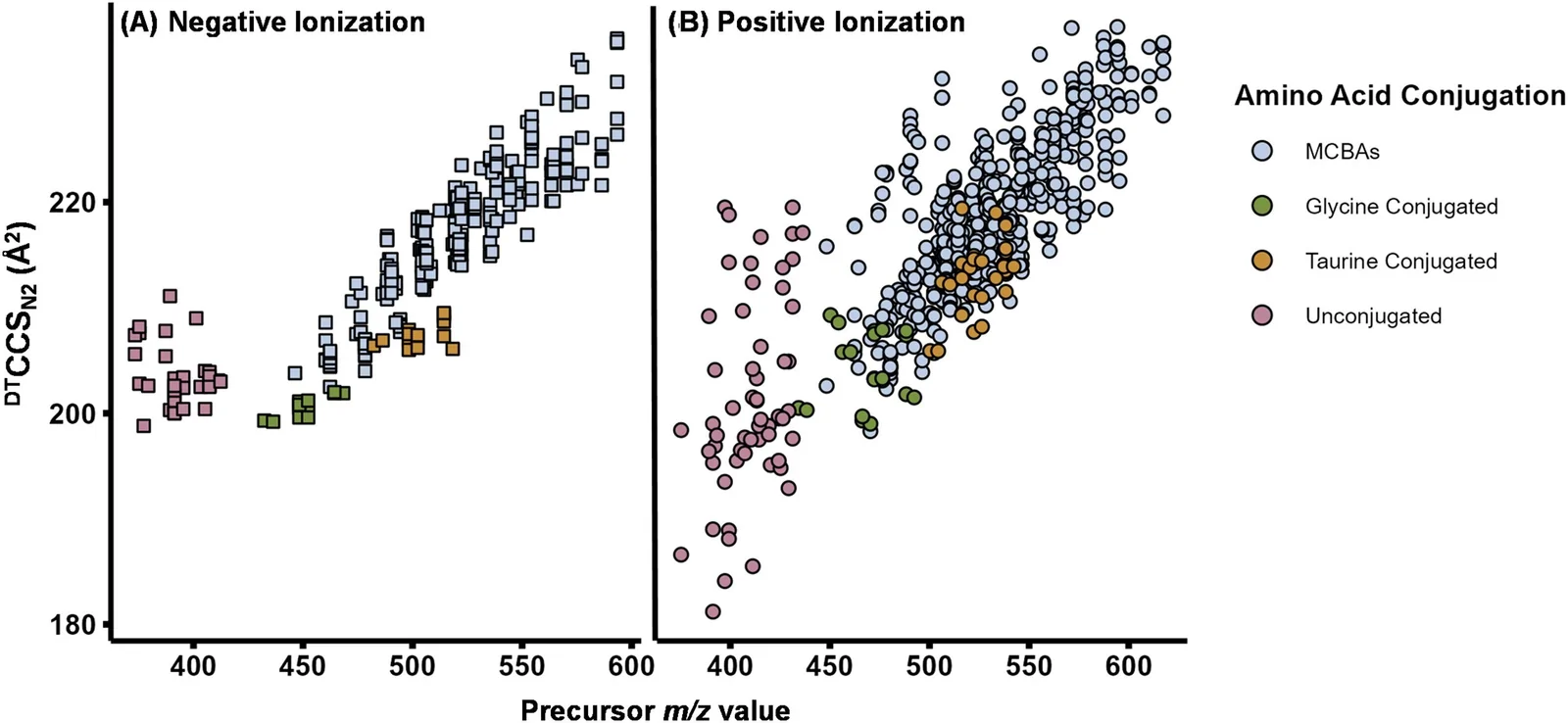
Bile acid library collision cross section (CCS) values versus mass-to-charge ratio (*m/z*) trends across ionization modes. (A) CCS values were measured for bile acids under negative ionization mode resulting in [M-H]^−^ ions and (B) positive ionization mode resulting in [M + H]^+^, [M + Na]^+^, and [M + NH_4_]^+^ ions. The bile acids are grouped by unconjugated, glycine and taurine conjugates, and MCBAs in the plots to illustrate the different trends.

## Discussion

In this work, we evaluated key analytical variables that influence bile acid detectability and annotation across complex biological matrices including stool, serum and plasma. Specifically, we compared extraction workflows, assessed chromatographic conditions and their practical trade-offs in separation performance and robustness, and evaluated ionization behavior across polarities.

Across extraction conditions, unconjugated bile acids were comparatively stable, whereas conjugated bile acids were more sensitive to workflow choices, underscoring the importance of solvent selection and handling steps when low-abundance conjugated species are of interest. For serum or plasma, we held the precipitation solvent system constant and focused on practical constraints such as available sample volume, showing that higher input improved detectability of lower-abundance conjugated bile acids under the tested conditions. Three LC methods were also evaluated for the bile acids with each showing advantages and limitations. Collectively, these comparisons highlight that analytical conditions depend on the matrix and study goals, and that documenting solvent composition, dilution strategy, and input amounts is important for reproducible bile acid profiling.

A multidimensional LC−IMS−MS reference library for 280 unique bile acids that integrate structures, LC retention time, accurate mass, experimental CCS values across ionization modes, and annotated ion forms and adduct types was also created in this work. The library includes 343 deprotonated features in negative mode and 665 features in positive mode spanning common adduct forms (e.g. protonated, sodiated, and ammoniated). By providing orthogonal separation information alongside curated feature expectations, this resource improves discrimination of structurally similar bile acids, including isomers and microbially conjugated species, and supports more confident annotation in complex samples. Together, the evaluated workflows and accompanying reference library provide a foundation for more reproducible and comprehensive bile acid profiling and support future investigations into bile acid diversity, host-microbiome interactions, and microbiome-associated metabolism.

## Supplementary Material

qvag039_Supplemental_Files

## Data Availability

All data from this study are publicly available. The raw mass spectrometry data (“.d” files) have been deposited in the MassIVE repository (https://massive.ucsd.edu) under accession number [MSV00096436]. The Skyline spectral library and processed results are available on Skyline Panorama (https://panoramaweb.org/PkMTb5.url). The bile acid library data, including LC−IMS−MS spectra, retention times, and CCS values, have been deposited in Zenodo (https://doi.org/10.5281/zenodo.18675023) and the structures and CCS values added to PubChem (see data subsets under the Baker Lab source https://pubchem.ncbi.nlm.nih.gov/source/25763 and CCS classification at https://pubchem.ncbi.nlm.nih.gov/classification/#hid=124). Protocols for bile acid extraction, including SOPs, are available in Supplemental Information.
