# Supplementary material for: Unlocking the bile acid universe: advanced workflows and a multidimensional library of 280 unique species
Source: Sustain Microbiol. 2026 Sep 15;3(4):qvag039. doi: 10.1093/sumbio/qvag039 (PMC13637491; doi:10.1093/sumbio/qvag039)
Supplement: Supplementary file 1 [file qvag039_supplemental_files.zip › Supplemental_Information_Clean_vs2.pdf]

Supporting Information

Unlocking the Bile Acid Universe: Advanced Workflows and a Multidimensional Library of 280 Unique Species

Authors

Guozhi Zhang[1], Emily C. Vincent[1], Sadie M. Disselkoen[1], James N. Dodds[1], Quentin DuVal-Smith[1], Abubaker Patan[2], Ipsita Mohanty[2], Victoria Deleray[2], Jian Zhang[3], Paul A. Thiessen[3], Evan E. Bolton[3], Emma L. Schymanski[4], Pieter C. Dorrestein[2], Casey M. Theriot[5], Erin S. Baker[1*]

Affiliations

[1]Department of Chemistry, University of North Carolina at Chapel Hill, Chapel Hill, NC, 27514, USA

[2]Collaborative Mass Spectrometry Innovation Center, Skaggs School of Pharmacy and Pharmaceutical Sciences, Departments of Pharmacology and Pediatrics, University of California at San Diego, La Jolla, California 92093, USA

[3]National Center for Biotechnology Information, National Library of Medicine, National Institutes of Health, Bethesda, MD 20894, USA

[4]Luxembourg Centre for Systems Biomedicine, University of Luxembourg, 6 Avenue du Swing, 4367, Belvaux, Luxembourg

[5]Department of Population Health and Pathobiology, North Carolina State University, Raleigh, NC, 27607, USA

*Corresponding author: Erin S. Baker (erinmsb@unc.edu)

Table of Contents

Cover Page and Table of Contents **S1-S2**

Supplemental Methods **S3-S14**

Standard Preparation **S3**

Instrumental Analysis **S3-S5**

Optional Quantification Guidance **S5-S6**

Data Analysis **S6-S7**

Bile Acid Extraction from Solid Matrices **S8-S11**

Bile Acid Extraction from Liquid Matrices **S12-S14**

LC Conditions and Recommended Run Order **S10**

Optional Quantification Guidance **S11-S12**

Supplemental Figures **S15-S18**

Figure S1. Stool extraction for glycine-conjugated bile acids **S15**

Figure S2. Serum and plasma extraction for glycine-conjugated bile acids **S16**

Figure S3. Serum and plasma extraction for bile acids under 1:4 (v/v) extraction conditions .. **S17**

Figure S4. Higher-dilution extraction condition 1:8 (v/v) for bile acids **S18**

Figure S5. Representative collision cross section (CCS) distributions for bile acid features detected in positive-ion mode **S18**

References **S19**

Supplemental Methods

Standard Preparation

Bile Acid Standard Preparation. Bile acid standards and mixtures were purchased from Cayman Chemical (Ann Arbor, MI) and Cambridge Isotope Laboratories (Tewksbury, MA) **(Supplemental Information)**. In addition to the Cayman and CIL standard mixtures, 22 individual unlabeled standards (^{12}C) were purchased from Cayman Chemical and synthetic bile acids were received from the Dorrestein lab and most can now be obtained from BileOmix, while others are part of a legacy collection from the late Alan Hoffmann and provided by Lee Hagey(Gentry et al. 2024; Patan et al. 2025). The standards were then used to populate the LC-IMS-MS reference library and support bile acid annotation (**Tables S1-S3**). Powdered standards were reconstituted in 1000 µL methanol to prepare an individual stock solution. Stocks were vortex-mixed until fully dissolved and stored for subsequent analyses. Individual unlabeled bile acid standards from Cayman were supplied in variable quantities, and accurately weighing sub-milligram amounts is prone to large errors. Therefore, each individual standard was reconstituted in 500 µL methanol to generate a stock standard in solution, vortex-mixed until fully dissolved, and stored at -20°C to minimize degradation. Because bile acids differ substantially in solubility and electrospray ionization (ESI) efficiency, the concentrations required to achieve comparable on-column signal vary by analyte. Accordingly, individual standards were prepared across a broad concentration range (0.5-100 mg/mL) to obtain measurable signal for each compound under the tested LC-IMS-MS conditions. The Cayman Bile Acids MaxSpec® Discovery Mixture (18 bile acids, catalog number 33505) was also utilized for LC method optimization and received in methanol and used without further preparation.

Internal Standard Preparation. A ^{13}C-labeled bile acid internal standard mixture was used as an extraction process control to monitor extraction consistency, instrument stability, and confirm successful extraction. The mixture was prepared by combining two CIL bile acid mixes: labeled conjugated bile acid mix (MSK-BA1-1) and a labeled unconjugated bile acid mix (MSK-BA2-1). Each vial was reconstituted in 1000 µL of a 1:1 methanol/water mixture, and the solutions were combined to yield a 2000 µL internal standard mix with an approximate concentration of 50 µM per bile acid (0.02 mg/mL MSK-BA1-1 and 0.025 mg/mL MSK-BA2-1). This internal standard was spiked into samples prior to extraction to monitor instrumental/extraction performance but was not used for signal normalization or quantification in the analyses reported here.

Instrumental Analysis

Flow Injection Analysis (FIA). For the library, some of the stock standard solutions were diluted in 50:50 (v/v) methanol:nanopure water for MS analysis with an Agilent 6560 IMS-QTOF mass spectrometry instrument. FIA (direct injection) was used to rapidly evaluate ionization behavior and IMS features for each standard in both ionization modes. For FIA, 0.01-0.2 µL of standard solution was injected in triplicate using a 50:50 mixture of mobile phase A (water + 5 mM ammonium acetate) and mobile phase B (50:50 (v/v) methanol:acetonitrile) (mobile phase set 1). Data were acquired in both positive and negative ionization modes over *m/z* 50-1700.

LC Separation. Injection volume (1-6 µL) was adjusted based on concentrations of each bile acid standard to maintain comparable peak shapes. For extracts, a fixed injection volume of 6 µL was used. All samples were injected into an Agilent 1290 UPLC coupled to an Agilent 6560 IMS-QTOF mass spectrometry instrument. All extracts and standards were evaluated in negative ESI mode, and positive mode was also

used for bile acid standards for library building. Detailed mobile phase compositions, and gradient programs are provided in **Table S7**.

The LC separation for the stool, serum, and plasma extracted samples were performed using a Restek Raptor C18 column (1.8 µm, 50 × 2.1 mm; Restek, catalog number 9304252), coupled with a ZORBAX Eclipse Plus C18 guard column (2.1 mm, 1.8 µm; Agilent Technologies, catalog number 821725-901). The guard column was used to protect the main analytical column from sample matrix components in biological samples and enhance reproducibility and separation consistency across analyses. The Restek column and neutral mobile phase were chosen based on their previous application in bile acid separations(Stewart et al. 2023), serving as a baseline condition for method comparisons.

For bile acid standards, three different LC methods were evaluated using both the Restek Raptor C18 column and the Waters ACQUITY Premier CSH C18 column (1.7 µm, 2.1 × 100 mm; Waters Corporation, catalog number 186009461). A total of three mobile phase sets, consisting of both neutral pH and acidic pH conditions, were tested. The column temperature was maintained at 60 °C for both columns with a flow rate of 0.5 mL/min for the Restek Raptor column and 0.4 mL/min for the CSH column.

LC Conditions and Recommended Run Order are detailed below:

Mobile Phases

- Method 1:
  - Mobile Phase A1: Water + 5 mM ammonium acetate
  - Mobile Phase B1: 50:50 (v/v) methanol:acetonitrile
- Method 2:
  - Mobile Phase A2: Water + 0.1% (v/v) formic acid
  - Mobile Phase B2: 50:50 (v/v) methanol:acetonitrile + 0.1% (v/v) formic acid
- Method 3:
  - Mobile Phase A3: Water + 0.1% (v/v) formic acid
  - Mobile Phase B3: Acetonitrile + 0.1% (v/v) formic acid

LC Columns and Operating Conditions

- Methods 1 and 2:
  - Restek Raptor C18 (1.8 µm, 50 × 2.1 mm, Catalog No. 9304252)
  - Flow rate: 0.5 mL/min
  - Column temperature: 60°C
  - Mobile phases for Methods 1 and 2

- Method 3:
  - Waters ACQUITY Premier CSH C18 (1.7 µm, 2.1 × 100 mm, Catalog No. 186009461)
  - Flow rate: 0.4 mL/min
  - Column temperature: 60°C
  - Mobile phase: set3

LC Gradients

- Detailed gradients are provided in **Table S4**

Calibration. Prior to any LC-IMS-MS analyses, mass calibration and CCS calibration were performed using the Agilent ESI-L Low Tuning Mix (Santa Clara, CA; catalog number G1969-85000), ensuring accurate mass calibration and precise CCS measurements.

IMS-MS Acquisition. The Agilent 6560 IM-quadrupole time-of-flight (QTOF) MS instrument was used to acquire the IMS and MS data in the experiment. Following chromatographic separation and ESI, the ions traveled through a single-bore glass capillary, where they were focused into a high-pressure funnel and accumulated in a trap funnel. The ions were then pulsed into a 78 cm IMS drift tube containing 3.95 Torr of nitrogen drift gas, using Hadamard transform multiplexing(Prost et al. 2014). A 4-bit pseudo-random pulsing sequence was applied for packet ejection from the trapping funnel, with a trap fill time of 3.9 ms and a release time of 150 µs. After traversing the drift tube, the ions were refocused in a rear ion funnel and analyzed using the QTOF over a range of 50-1700 *m/z*. The ".d" files were then collected containing information on the LC, IMS and MS values for each ion detected in the experiments. Details of the MS parameter can be found in **Table S8.**

Recommend Sample Injection Sequence (Worklist).

Order	Sample	Note
1	Tune Mix	Agilent Tuning Mix (p/n G1969-85000)
2	Non-injection	Run the LC gradient without injection to equilibrate the system and assess baseline
3	ES/DB	Injection of the extraction solvent used for sample preparation
4	EB	Extraction blank, processed using the extraction workflow without biological sample
5	MB	Method blank, LC-MS grade water processed identically to samples
6	Unlabeled BA	Unlabeled standard mix (#2 in Materials), injected at an appropriate concentration for system suitability
7	Samples	Randomized study samples; include NIST reference material and/or calibration standards if absolute quantification is performed

<u>Optional Quantification Guidance</u>

The following guidance is provided as an optional framework for users who wish to implement relative or absolute quantification. Quantification procedures were not a focus of the current study and are not required to reproduce the extraction workflow described here.

Relative Quantification Using Internal Standards or Absolute Quantification Using External Calibration

- Internal standards (recommended for all analysis):

  Stable isotope labeled internal standards (ISD) are spiked into each sample prior to extraction to normalize instrument response, monitor extraction performance, and assess matrix effects across the batch. This approach supports relative comparisons of bile acid abundances between samples.

- External calibration (for absolute quantification):

  For absolute quantification, prepare an external calibration curve using the unlabeled standard and spike with a fixed volume of the internal standard (ISD). Calibration curves are generated by plotting the peak area ratio of each unlabeled bile acid to the matched deuterated internal standard versus the nominal concentration of the native analyte, and sample concentrations are calculated using the same area-ratio approach. Calibration standards should be processed using the same extraction and cleanup workflow as study samples to minimize bias from matrix effects and sample preparation.

Steps for Preparation and Processing Calibration Curve Standards

1. Recommended Standard Concentrations (unlabeled, #2 in Materials):
    - Prepare calibration standards at the following concentrations: 0, 0.16, 0.8, 4, 20, 50, 100 µM.

Note: Concentration points may be adjusted, provided the range spans the expected sample concentrations.

2. Preparation of Calibration Standards:
    - Aliquot 100 µL of each calibration standard into a 1.5 mL microcentrifuge tube.
    - Spike each tube with a fixed volume of internal standard (ISD; #1 in Materials), typically 2 µL (range 2-5 µL depending on ISD concentration and expected instrument response).
    - Mix thoroughly.
3. Processing of Calibration Standards:
    - Process each calibration standard using the same extraction, mixing, and filtration steps as the study samples to ensure equivalent matrix handling and to minimize bias from sample preparation.
    - Calibrators should be randomized within the batch and injected periodically as needed to monitor drift.
    - Matrix-matched calibrators are recommended when feasible to account for matrix effects.

Extraction Blank and Method Blank

Process all the blanks using the same steps as experimental samples to ensure comparable handling.

- Extraction Blank:
  Follow the extraction procedure using an empty extraction tube (no biological sample). Add extraction buffer according to the procedure, but do not add internal standard.

- Method Blank:
  Follow the Stool Sample Extraction Procedure, Follow the extraction procedure, substituting LC-MS grade water (#6 in Materials) for the biological sample. Add internal standard and extraction buffer as specified in the procedure.

Data Analysis

CCS Calculation. Drift times were extracted from raw Agilent ".d" files using Agilent MassHunter IM-MS Browser 10.0. CCS values were then calculated using the single field(Stow et al. 2017). For all reported analytes, *m/z* mass errors were under 10 ppm and CCS the percent relative standard deviation (%RSD) across replicates was below 0.3%.

Demultiplexing. Raw ".d" files were demultiplexed using PNNL-Preprocessor 4.0 with a moving average of 3, a minimum pulse coverage of 100%, and a signal intensity threshold of 20 counts, generating DeMP.d files. Demultiplexed data were analyzed in Agilent MassHunter IM-MS Browser 10.0 to extract drift times. CCS values were then calculated using the single-field approach in a Microsoft Excel workbook, which

applies the single-field calibration parameters to convert drift time to CCS. Microsoft Excel was also used to summarize mass accuracy (ppm error of measured *m/z*) and CCS precision across replicate injections. For the standards reported in the manuscript, m/z errors were under 10 ppm and CCS %RSD across replicates was below 0.3%.

Skyline Targeted Bile Acid Analyses. Skyline 23.1 (MacCoss Lab, Seattle, WA, release date 9/24/23), was used for compiling retention time information from standards and targeted peak integration to annotate bile acids in the extraction samples. Bile acid standards and mixtures acquired using the three LC conditions were imported into Skyline and manually annotated based on the reference library developed in this study. Retention times for annotated targets were exported to Microsoft Excel and summarized in **Tables S1-S2**. Deuterated standards are listed in both the quantitation and deuterated sections of Tables S1 and S2 for ease of import into Skyline. For extraction analyses, stool, serum, and plasma ".d" data were imported into Skyline, and peaks for bile acids and MCBAs were integrated using the same target library list. Peak area under the curve (AUC) for each bile acid and MCBA, along with mass errors, was exported to Microsoft Excel for further analysis. Data points with mass errors exceeding 10 ppm were excluded from processing.

Bile Acid Extraction SOPs

This Standard Operating Procedure (SOP) provides general guidance for extracting bile acids from solid and liquid biological matrices for downstream analysis by LC-IMS-MS (and conventional LC-MS, where applicable). The workflow is designed to be adaptable across sample types, spanning solid matrices such as feces, cecal contents, liver, and other tissues, and liquid matrices such as serum, plasma, bacterial culture supernatants, and other biofluids. The procedures use matrix-appropriate homogenization or protein-precipitation strategies, internal standardization, and optional external calibration for absolute quantification. Key parameters (e.g., solvent composition, solvent-to-sample ratio, internal standard concentration/spike volume, and clarification/filtration conditions) should be optimized as needed to accommodate matrix or sample dependent properties (e.g., expected bile-acid abundance) and the analytical objectives. Chromatographic conditions and a recommended sample injection sequence are provided to support method implementation and batch quality control.

Information for Bile Acid Extraction from Solid Biological Matrices including Stool

The extraction procedure for bile acids from solid biological matrices follows previously established protocols(McMillan et al. 2024) and the SOP is given below. Briefly, approximately 50 mg of a control human stool sample(McMillan et al. 2024)was aliquoted into eight 2 mL microtubes containing 2.38 mm metal beads (Omni International, Kennesaw, GA, USA; catalog number 19-620) to compare four different extraction solvent conditions. Due to sample limitations, each extraction condition was performed in duplicate to ensure reproducibility. The exact stool conditions and masses used for each replicate are provided in **Tables S4-S5**, and the extraction conditions are noted below.

Pre-extraction Buffer Preparation. Four different HPLC grade organic solvents were evaluated for stool extraction: ethanol (Sigma-Aldrich, St. Louis, MO, catalog number 226557), methanol (Fisher Scientific, catalog number 67-56-1), acetonitrile (Fisher Scientific, Hampton, NH, catalog number 75-05-8), and a 1:1 (v/v) mixture of acetonitrile (ACN) and methanol (MeOH) **(Table S4)**. The four solvents were selected based on their effectiveness in extracting bile acids of varying hydrophobicity, as supported by previous studies(McMillan et al. 2024). Each pre-extraction buffer was prepared by diluting a 100 mM potassium phosphate monobasic buffer (Sigma-Aldrich, catalog number 7778-77-0) 1:5 (v/v) with water to achieve a 20 mM aqueous phosphate solution. Pre-extraction buffer was then prepared by combining 60 mL of the 20 mM phosphate solution with 340 mL designated organic solvent to obtain a 15:85 (v/v) aqueous:organic ratio (total volume 400 mL) and a final phosphate concentration of 3 mM. For the 1:1 acetonitrile:methanol condition, the 340 mL organic fraction is comprised of 170 mL acetonitrile and 170 mL methanol. Each pre-extraction buffer was added to the stool samples at a ratio of 8 µL per mg of sample (*e.g.,* 400 µL for 50 mg) to account for minor variations in sample mass.

Sample Extraction and Processing. Briefly, prior to extraction, a fixed volume of the ^{13}C-labeled bile acid internal standard mix was added to each tube. Samples in pre-extraction buffer were homogenized at room temperature using a Fisherbrand™ Bead Mill 24 Homogenizer (Fisher Scientific, catalog number 15-340-163) at 2.1 m/s for 2 min in a single cycle to ensure complete disruption. The homogenized samples were centrifuged at 13,000 × g for 10 min at 4°C. The resulting supernatant from each extraction condition was divided into three fractions: 100 µL was mixed 1:1 with methanol, 50 µL was mixed 1:4 with methanol, and 50 µL was mixed 1:4 with the same organic solvent (without phosphate) used in the corresponding pre-extraction step. Each mixture was vortexed at 600 rpm for 20 min at room temperature to ensure thorough extraction. The extracted samples were then filtered using MilliporeSigma PTFE membrane spin filters (0.2

µm pore size, catalog number UFC30LG25), and a 60 µL aliquot of the filtrate was transferred to Agilent LC vials (Agilent Technologies, catalog number 5188-6591) for analysis.

SOP for Bile Acid Extraction from Solid Biological Matrices including Stool

Materials and Reagents

1. Make Internal Standard (ISD):

- Concentration**:** 50-200 µM solution in 50% methanol (MeOH), depending on experimental needs
- Catalog Numbers**:** MSK-BA1 and MSK-BA2 from Cambridge Isotope Laboratories
- Preparation (as the example of 200 µM final concentration)**:**
  i. Add 250 µL of 50:50 (v/v) methanol:nanopure water to each ISD vial.
  ii. Vortex until the contents are fully dissolved.
  iii. Centrifuge the vials at 500 x g for 1 minute.
  iv. Combine the contents of both vials into a single ambient container from Agilent. (Part number: 8010-0543)

2. Make Unlabeled Standard:

- Concentration**:** 100 µM solution in 50% methanol (MeOH)
- Catalog Numbers**:** MSK-BA1-US-1 and MSK-BA2-US-1
- Preparation**:**
  i. Add 500 µL of 50:50 (v/v) methanol:nanopure water water to each vial.
  ii. Vortex until the contents are fully dissolved.
  iii. Centrifuge the vials at 500 x g for 1 minute.
  iv. Combine the contents of both vials into a single ambient vial from Agilent. (Part number: 8010-0543)

Note: A 100 µM stock is recommended to support calibration that spans higher bile acid abundances. If lower concentration standards are needed, they can be prepared by serial dilution from the 100 µM stock using 50:50 (v/v) MeOH:water. Alternatively, a commercial bile acid mixture at lower concentration (e.g., Bile Acids MaxSpec® Discovery Mixture, Cayman Chemical) may be used directly when the expected concentration range does not require high-end coverage.

3. Prepare an Extraction Solvent: 1:1 acetonitrile (ACN): methanol (MeOH) with phosphate (Pre-extraction buffer)

- Composition: 1:1 (v/v) acetonitrile (ACN):methanol (MeOH) containing phosphate (final 3 mM)
- Stock Potassium Phosphate Buffer: 100 mM (Catalog No.: 7778-77-0)
- Preparation**:**
  i. Dilute the 100 mM phosphate stock 1:5 (v/v) with water to obtain 20 mM phosphate.
  ii. Mix 60 mL of 20 mM phosphate buffer with 340 mL organic solvent (170 mL MeOH + 170 mL ACN) to yield 400 mL of pre-extraction buffer with 3 mM phosphate.

4. NIST Standard for Reference

- NIST 1950 Metabolites in human plasma
- NIST 909C Human serum

5. LC Vials: Agilent glass vials with inserts

- Catalog Number: 5188-6591 from Agilent

6. Optima ™LC-MS Grade Solvents for Mobile Phases and Blanks

- Methanol: Catalog Number: 67-56-1
- Acetonitrile: Catalog Number: 75-05-8
- Water: Catalog Number: 7732-18-5
  **All solvents purchased from Fisher Chemical, Pittsburgh, PA*

7. Sample Tubes for Extraction

- 1.5 mL microcentrifuge tube: Catalog Number: 3451 (Thermo Scientific)
- 2 mL beadmill tube: Catalog Number: 19-620 (Revvity)
- PTFE spin filter tube: Catalog Number: UFC30LG25 (Millipore)

Extraction Procedure for Solid Matrices:

1. Internal Standard Addition

Add 2 µL of 200 µM ISD solution (#1 in Materials) to each 2 mL beadmill tube (#7 in Materials) containing 50-100 mg solid sample (e.g., stool).

2. Sample-to-solvent Weight Normalization

Add extraction buffer (#3 in Materials) at 8 µL per 1 mg sample (8:1 µL/mg).

Example: 75 mg sample, add 600 µL extraction buffer.

3. Homogenization

Homogenize using a Fisherbrand™ Bead Mill 24 Homogenizer (Hampton, NH, USA; catalog number 15-340-163) at 2.1 m/s for 2 minutes in a single cycle

Note: This setting is typically sufficient for fecal and cecal samples. For harder tissues (e.g., liver or other solid organs), two homogenization cycles may be required. Allow a brief pause, placing tubes on ice between cycles, to minimize sample heating.

4. Centrifugation

Centrifuge the samples at 10,000 × g for 15 min or 13,000 × g for 10 min at 4°C.

Note: For solid matrices, lower centrifugation speeds may be preferable when tube balancing is limited by sample mass variability.

5. Extraction

Transfer 100 µL of supernatant into a fresh 1.5 mL microcentrifuge tube and add 100 µL MeOH. Mix briefly.

6. Mixing

Mix at 600 rpm for 20 min at room temperature using an Eppendorf MixMate® (Catalog No.: 5353000529).

7. Filtration

Transfer the full 200 µL to a PTFE spin filter tube and centrifuge at 10,000 × g for 1 min at 4°C. Collect the filtrate.

8. Preparation for LC-MS Analysis

Transfer 60 µL filtrate into labeled LC vials (#5 in Materials) for LC–IMS–MS analysis (or LC–MS, where applicable).

Note: If needed to improve solvent compatibility with the initial LC conditions, the extract may be diluted with nanopure water. In our workflow, extracts were injected without dilution and acceptable chromatography was obtained; dilution may be used as an optimization step if peak shape or retention is suboptimal. Record any dilution factor applied, as this decreases analyte concentration.

Information for Bile Acid Extraction from Liquid Biological Matrices including Serum and Plasma

Bile acids were extracted from human control serum and plasma samples, and NIST serum 909C and NIST plasma 1950 using a 1:1 (v/v) acetonitrile:methanol with a detailed supplemental SOP and **Table S6**. These extraction solvents were added at a 1:4 ratio to two different sample volumes of serum and plasma (200 µL and 50 µL) to compare high and low sample quantities, and performed in triplicate (n=3), resulting in 24 samples for the serum and plasma including NIST reference (*e.g.,* 4 matrices × 2 volumes × 3 replicates). Additionally, a 1:8 extraction ratio was also evaluated using 50 µL of the sample, but this was not replicated due to sample limitations.

Sample Extraction and Processing. A fixed volume of the ^{13}C-labeled bile acid internal standard mix was added to each sample above. Samples were vortexed at 2,000 rpm for 5 min at room temperature, followed by sonication on ice for 15 min to enhance bile acid extraction efficiency. Samples were then incubated overnight at -20°C, allowing for protein precipitation. This step was included to ensure consistent extraction efficiency across different sample volumes. The following day, samples were centrifuged at 13,000 × g for 10 min at 4°C using an Eppendorf 5810R centrifuge. A 100 µL aliquot of the supernatant was transferred into a clean 2.0 mL Eppendorf tube (MilliporeSigma, catalog number 30123620) for further analysis.

SOP for Bile Acid Extraction from Liquid Biological Matrices including Serum and Plasma

Materials and Reagents

1. Make Internal Standard (ISD):

- Concentration**:** 50-200 µM solution in 50% methanol (MeOH), depending on experimental needs
- Catalog Numbers**:** MSK-BA1 and MSK-BA2 from Cambridge Isotope Laboratories
- Preparation (as the example of 200 µM final concentration)**:**
  i. Add 250 µL of 50:50 (v/v) MeOH:watermethanol:nanopure water to each ISD vial.
  ii. Vortex until the contents are fully dissolved.
  iii. Centrifuge the vials at 500 x g for 1 minute.
  iv. Combine the contents of both vials into a single ambient container from Agilent. (Part number: 8010-0543)

2. Make Unlabeled Standard:

- Concentration**:** 100 µM solution in 50% methanol (MeOH)
- Catalog Numbers**:** MSK-BA1-US-1 and MSK-BA2-US-1
- Preparation**:**
  i. Add 500 µL of 50:50 (v/v) MeOH:watermethanol:nanopure water water to each vial.
  ii. Vortex until the contents are fully dissolved.
  iii. Centrifuge the vials at 500 x g for 1 minute.
  iv. Combine the contents of both vials into a single ambient vial from Agilent. (Part number: 8010-0543)

Note: A 100 µM stock is recommended to support calibration that spans higher bile acid abundances. If lower concentration standards are needed, they can be prepared by serial dilution from the 100 µM stock

using 50:50 (v/v) MeOH:water. Alternatively, a commercial bile acid mixture at lower concentration (e.g., Bile Acids MaxSpec® Discovery Mixture, Cayman Chemical) may be used directly when the expected concentration range does not require high-end coverage.

3. Prepare an Extraction Solvent: 1:1 acetonitrile (ACN): methanol (MeOH)

- Composition: 1:1 (v/v) acetonitrile (ACN):methanol (MeOH)

4. NIST Standard for Reference

- NIST 1950 Metabolites in human plasma
- NIST 909C Human serum

5. LC Vials: Agilent glass vials with inserts

- Catalog Number: 5188-6591 from Agilent

6. Optima ™LC-MS Grade Solvents for Mobile Phases and Blanks

- Methanol: Catalog Number: 67-56-1
- Acetonitrile: Catalog Number: 75-05-8
- Water: Catalog Number: 7732-18-5
  **All solvents purchased from Fisher Chemical, Pittsburgh, PA*

7. Sample Tubes for Extraction

- 1.5 mL microcentrifuge tube: Catalog Number: 3451 (Thermo Scientific)

Extraction Procedure for Liquid Matrices:

1. Internal Standard Addition

Add 2 µL of 200 µM ISD solution (#1 in Materials) to each 1.5 mL microcentrifuge sample tube(#7 in Materials).

2. Sample Preparation

Put 50-100 µL of liquid sample (e.g., plasma/serum) to the tube.

3. Solvent Addition

Add 400 µL of extraction solvent (1:1 ACN:MeOH; #3 in Materials) to achieve a 4:1 solvent-to-sample ratio (v/v).

4. Mixing and Sonication

i. Mix at 2,000 rpm for 5 min using an Eppendorf MixMate® (Catalog No.: 5353000529) at room temperature.

ii. Sonicate for 15 min in an ice bath to enhance extraction.

5. Cold Precipitation

Store the samples at -20°C overnight to promote protein precipitation and improve supernatant clarity.

Note: This step may be shortened (e.g., ≥1h at -20°C) depending on matrix protein content and throughput requirements.

6. Centrifugation

Centrifuge at 13,000 × g for 10 min at 4°C.

7. Supernatant Collection

Transfer 300 µL of the supernatant to a new 1.5 mL microcentrifuge tube (#7 in Materials).

8. Preparation for LC-MS Analysis

Transfer 60 µL of the supernatant into LC vials (#5 in Materials) for LC–IMS–MS analysis (or LC–MS, where applicable).

Note: If needed to improve solvent compatibility with the initial LC conditions, the extract may be diluted with nanopure water. In our workflow, extracts were injected without dilution and acceptable chromatography was obtained; dilution may be used as an optimization step if peak shape or retention is suboptimal. Record any dilution factor applied, as this decreases analyte concentration.

Supplemental Figures

Figure S1. Stool extraction for glycine-conjugated bile acids (glycine conjugated to ursodeoxycholic acid (GUDCA), lithocholic acid (GLCA), hyodeoxycholic acid (GHDCA), deoxycholic acid (GDCA), chenodeoxycholic acid (GCDCA), and cholic acid (GCA)). Bile acid extractions were performed on stool samples (n=2 biological replicates) using various pre-extraction buffers and second-step dilution conditions. Extraction conditions included four pre-extraction buffers (ethanol, methanol, acetonitrile, or 1:1 acetonitrile/methanol), followed by three dilution conditions: **A)** 1:1 dilution in methanol, **B)** 1:4 dilution in methanol, and **C)** 1:4 dilution in the matching pre-extraction solvent. Glycine-conjugated bile acids were analyzed by LC-IMS-MS. Signal intensity was measured for each condition, with results shown in the corresponding chromatograms and peak areas. Average percent relative abundance was calculated based on area under the peak ÷ total ion current × 100. This analysis was only tested in duplicate due to sample limitations.

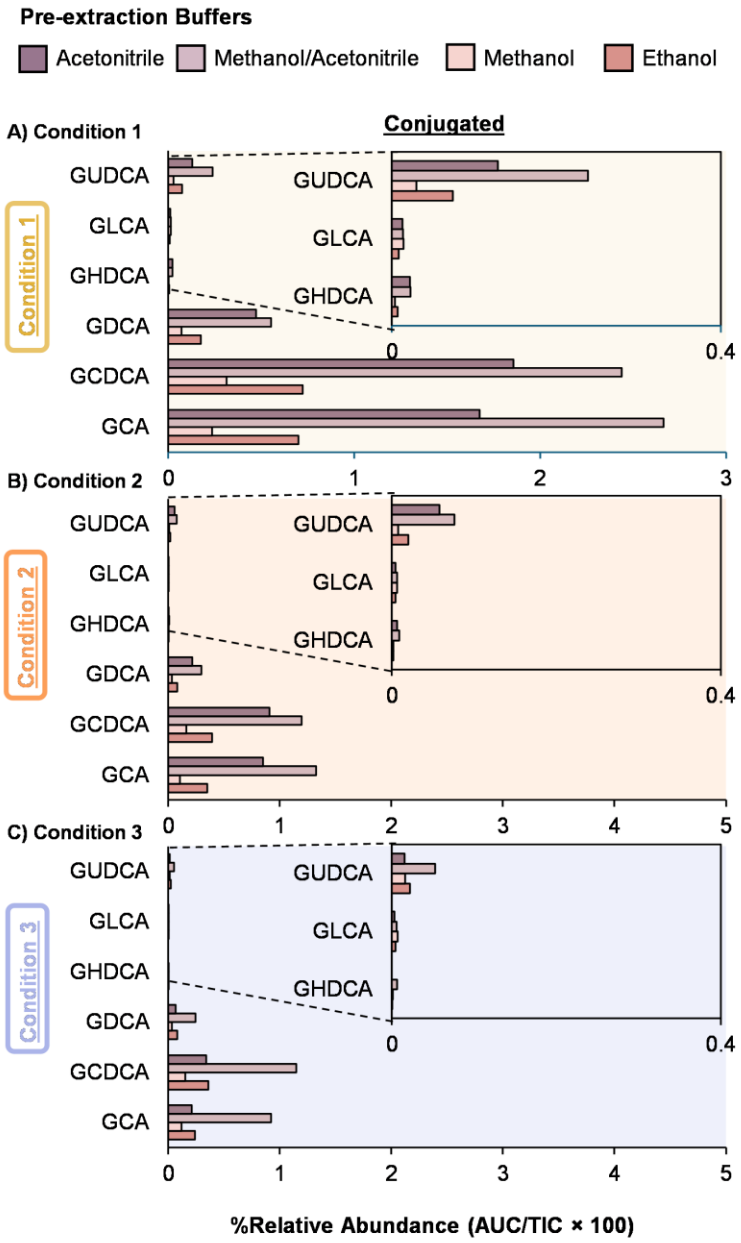

Figure S2. Serum and plasma extraction for glycine-conjugated bile acids (glycine conjugated to ursodeoxycholic acid (GUDCA), lithocholic acid (GLCA), hyodeoxycholic acid (GHDCA), deoxycholic acid (GDCA), chenodeoxycholic acid (GCDCA), and cholic acid (GCA)). Glycine-conjugated bile acids were extracted from human serum and plasma samples (n=3 biological replicates) using a 1:4 sample-to-solvent ratio with a 1:1 acetonitrile/methanol mixture. Two sample volumes were tested: **A)** 200 µL and **B)** 50 µL. Average percent relative abundance was calculated based on area under the peak ÷ total ion current × 100.

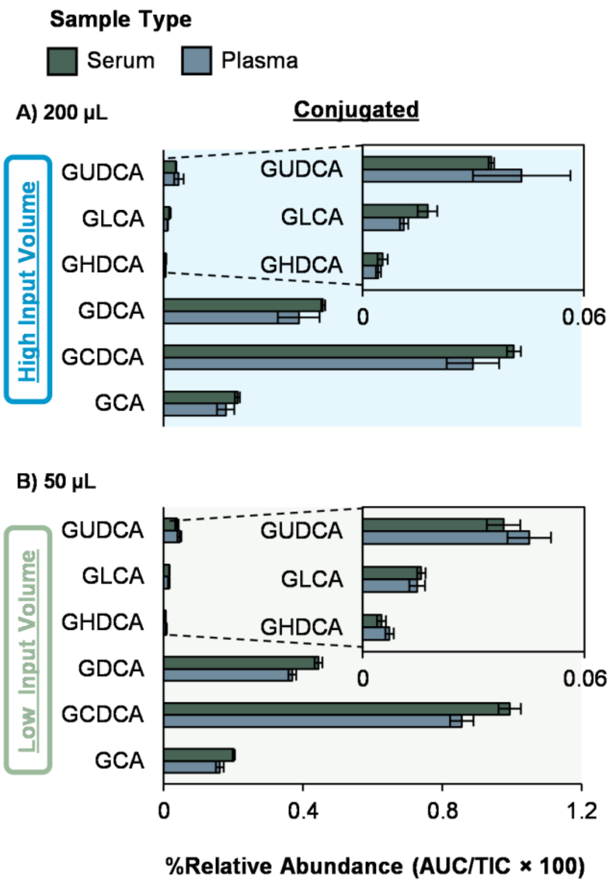

Figure S3. Serum and plasma extraction for bile acids under 1:4 (v/v) extraction conditions. NIST reference serum and plasma were extracted using a 1:4 solvent-to-sample ratio with a 1:1 acetonitrile:methanol mixture. Two sample volumes were tested: **A)** 200 µL and **B)** 50 µL. Unconjugated (left; lithocholic acid (LCA), deoxycholic acid (DCA), chenodeoxycholic acid (CDCA), hyodeoxycholic acid (HDCA), cholic acid (CA), and isolithocholic acid (isoLCA)), as well as their glycine-conjugated (middle) and taurine-conjugated (right) derivatives were analyzed. Percent relative abundance was calculated based on area under the peak ÷ total ion current × 100.

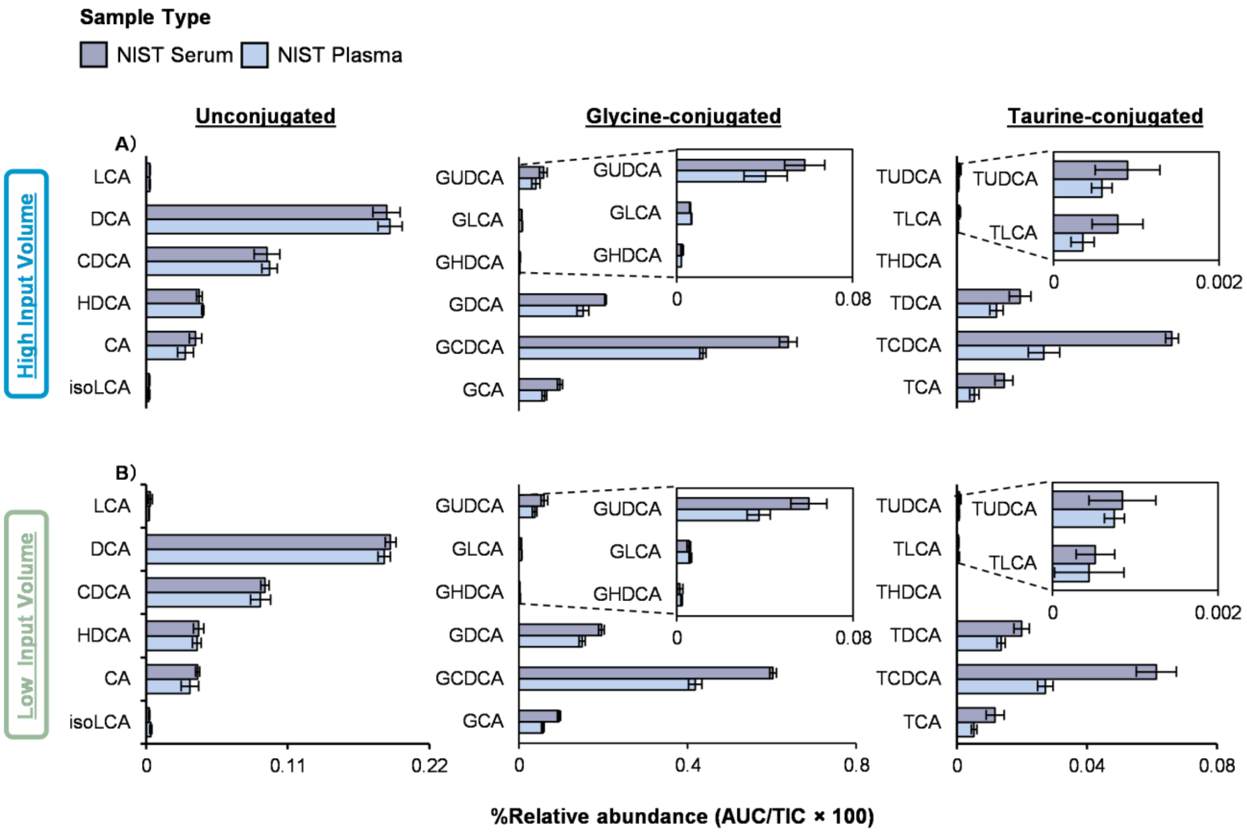

Figure S4. Higher-dilution extraction condition 1:8 (v/v) for bile acids. A higher-dilution extraction condition (1:8 sample-to-solvent ratio) was tested using 50 µL of each sample type: serum, plasma, NIST reference serum, and NIST reference plasma. **A)** Unconjugated bile acids (including lithocholic acid (LCA), deoxycholic acid (DCA), chenodeoxycholic acid (CDCA), hyodeoxycholic acid (HDCA), cholic acid (CA), and isolithocholic acid (isoLCA)), **B)** Glycine-conjugated bile acids, and **C)** Taurine-conjugated bile acids. This condition was evaluated without replication due to sample limitations.

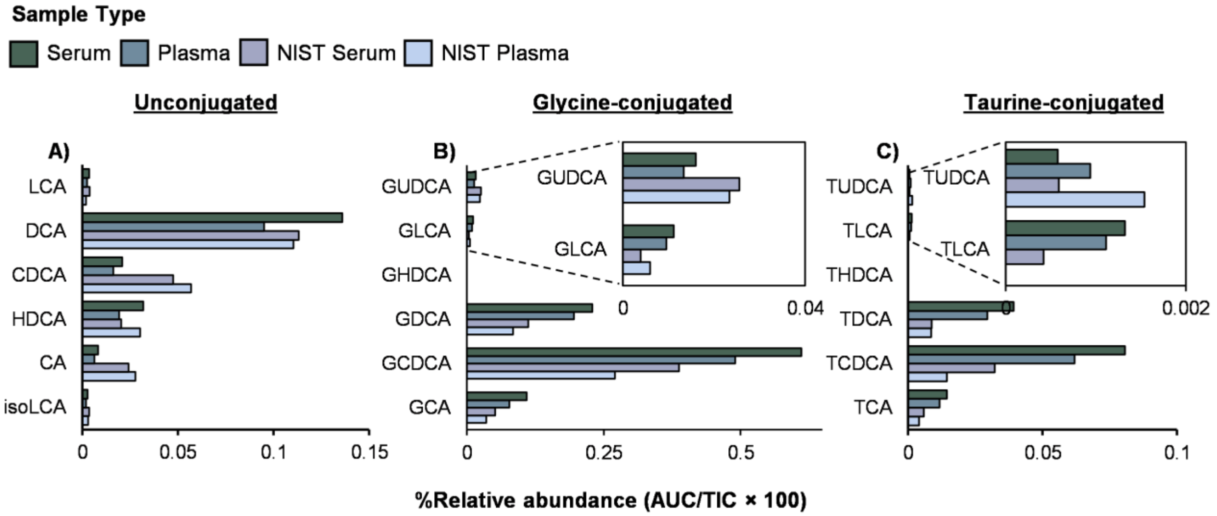

Figure S5. Representative collision cross section (CCS) distributions for bile acid features detected in positive-ion mode. CCS distributions are shown for common adducts, including **A)** $[M+H]^+$, **B)** $[M+Na]^+$, and **C)** $[M+NH_4]^+$.

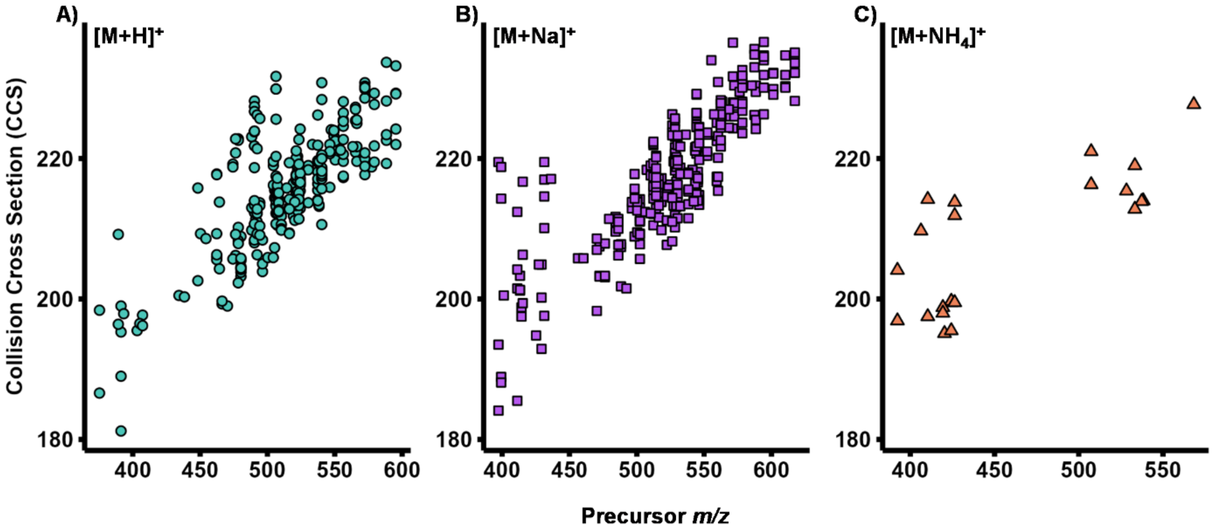

References

Gentry, E. C., et al. (2024), 'Reverse metabolomics for the discovery of chemical structures from humans', *Nature,* 626 (7998), 419-26.

McMillan, A. S., et al. (2024), 'Metagenomic, metabolomic, and lipidomic shifts associated with fecal microbiota transplantation for recurrent Clostridioides difficile infection', *mSphere,* 9 (10), e0070624.

Patan, A., et al. (2025), 'Charting the Undiscovered Metabolome with Synthetic Multiplexing', *bioRxiv*, 2025.11.18.689170.

Prost, S. A., et al. (2014), 'Detecting and removing data artifacts in Hadamard transform ion mobility-mass spectrometry measurements', *J Am Soc Mass Spectrom,* 25 (12), 2020-27.

Stewart, A. K., et al. (2023), 'Using Multidimensional Separations to Distinguish Isomeric Amino Acid-Bile Acid Conjugates and Assess Their Presence and Perturbations in Model Systems', *Anal Chem,* 95 (41), 15357-66.

Stow, S. M., et al. (2017), 'An Interlaboratory Evaluation of Drift Tube Ion Mobility-Mass Spectrometry Collision Cross Section Measurements', *Anal Chem,* 89 (17), 9048-55.
